# Mesenchymal Stem Cell Biodistribution, Migration, and Homing *In Vivo*

**DOI:** 10.1155/2014/292109

**Published:** 2014-03-11

**Authors:** Weian Zhao, Donald G. Phinney, Dominique Bonnet, Massimo Dominici, Mauro Krampera

**Affiliations:** ^1^Sue and Bill Gross Stem Cell Research Center, Chao Family Comprehensive Cancer Center, Department of Biomedical Engineering and Department of Pharmaceutical Sciences, University of California, Irvine, USA; ^2^Department of Molecular Therapeutics, The Scripps Research Institute, Jupiter, FL, USA; ^3^Haematopoietic Stem Cell Laboratory Cancer Research UK, London Research Institute, Lincoln's Inn Fields Laboratories, London, UK; ^4^Laboratory of Cell Biology and Advanced Cancer Therapies, Department of Medical and Surgical Sciences for Children & Adults, University Hospital of Modena and Reggio Emilia, Modena, Italy; ^5^Section of Hematology, Stem Cell Research Laboratory and Cell Factory, Department of Medicine, University of Verona, Policlinico G.B. Rossi, P.le L.A., Italy

Mesenchymal stem cells (MSCs) were originally defined by their capacity to differentiate into various connective tissue lineages as well as support hematopoiesis* in vitro *via the production of various cytokines, chemokines, and adhesion molecules [1, 2]. During the past decade, MSCs have been shown to exhibit angiogenic, trophic, anti-inflammatory, and immunomodulatory activity using a variety of experimental paradigms [3–6]. Together with their easy availability and amenability to large-scale expansion* ex vivo*, these properties have propelled MSC-based therapies to the forefront of regenerative medicine and immune regulatory cell therapy. Currently, MSCs from a variety of tissue sources are being evaluated in over 200 clinical trials for the treatment of a diverse array of disease indications. Completed Phase I and II clinical trials have reported statistically significant benefits in patients with steroid-resistant graft versus host disease [7], severe systemic lupus erythematosus [8], complex perianal fistulas [9], and ischemic cardiomyopathy [10]. However, not all trials have met their primary endpoint of efficacy and while many factors contribute to suboptimal patient outcomes, key among them are the molecular mechanisms that govern MSC engraftment, homing, and biodistribution* in vivo*. Indeed, despite rapid progress in describing the therapeutic potency of MSCs in experimental animal models of disease, progress in understanding their biodistribution and mechanism of action* in vivo* has been slow to develop. For example, robust methodologies to track the fate of MSCs* in vivo* are critical toward establishing their tissue tropism, duration of engraftment, and rates of clearance. In addition, the identification of endogenous factors that function as chemoattractants and repellents also plays critical roles in directing transplanted cells to sites of tissue injury. Moreover, a clearer understanding of the signaling axes that regulate MSC trafficking* in vivo* would provide a means to direct cells to specific tissue and organs, thereby increasing overall efficacy of MSC-based therapies. The latter may also provide a means to mobilize endogenous MSCs and enhance their regenerative and immune regulatory properties. Finally, cellular crosstalk and cell-to-cell interactions also likely affect the biodistribution and survival of exogenously administered MSCs, but scant information exists regarding these processes* in vivo*. In fact, it is a subject of debate whether MSCs localize to tissue due to passive entrapment in small vessels, particularly in lung, or if cells employ active mechanisms similar to leukocytes to home to specific tissues. Therefore, continued study into the mechanism that regulates trafficking of endogenous and transplanted MSCs will shed novel insight into basic MSC biology and lead to the development of more potent cell-based therapies.

We hope that the readers of this special issue will find it highly informative. The papers contained within it address many of the aforementioned issues including methods to track MSCs* in vivo*, mechanisms that mediate MSC migration and homing including within the CNS, and novel delivery methods to target cells to specific organs. This piece of information will serve as a useful resource with respect to current limitations in the field and provide insights as to how to improve current methods to achieve more beneficial outcomes for MSC-based therapies.

*Weian Zhao* *Weian Zhao* *Donald G. Phinney* *Donald G. Phinney* *Dominique Bonnet* *Dominique Bonnet* *Massimo Dominici* *Massimo Dominici* *Mauro Krampera* *Mauro Krampera*
